# Mapping Research Conducted on Long-Term Care Facilities for Older People in Brazil: A Scoping Review

**DOI:** 10.3390/ijerph18041522

**Published:** 2021-02-05

**Authors:** Patrick Alexander Wachholz, Deborah Cristina De Oliveira, Kathryn Hinsliff-Smith, Reena Devi, Paulo José Fortes Villas Boas, Victoria Shepherd, Alessandro Ferrari Jacinto, Helena Akemi Wada Watanabe, Adam Lee Gordon, Natalia Aquaroni Ricci

**Affiliations:** 1 Botucatu Medical School, São Paulo State University, Botucatu SP-01049-010, Brazil; paulo.boas@unesp.br; 2 Department of Psychiatry, Universidade Federal de São Paulo, São Paulo SP-04021-001, Brazil; oliveiradc.phd@gmail.com; 3 Faculty of Health and Life Sciences, Institute of Health, Health Policy and Social Care Research, De Montfort University, Leicester LE1 9BH, UK; kathryn.hinsliff-smith@dmu.ac.uk; 4 Faculty of Medicine and Health, School of Healthcare, University of Leeds, Leeds LS2 9J, UK; r.devi@leeds.ac.uk; 5 Centre for Trials Research, Cardiff University, Cardiff CF14 4YS, UK; shepherdVL1@cardiff.ac.uk; 6 Geriatric and Gerontology Discipline, Universidade Federal de São Paulo, São Paulo SP-04021-001, Brazil; alessandrojacinto@uol.com.br; 7 School of Public Health, University of São Paulo, São Paulo SP-01246-904, Brazil; hwatanab@usp.br; 8 Medical School, Royal Derby Hospitals, University of Nottingham, Nottingham DE22 3NE, UK; adam.gordon@nottingham.ac.uk; 9 NIHR Applied Research Collaboration—East Midlands (ARC-EM), Nottingham DE22 3NE, UK; 10 Master’s and Doctoral Programs in Physical Therapy, Universidade Cidade de São Paulo, São Paulo SP-03071-000, Brazil; natalia_ricci@hotmail.com

**Keywords:** older adults, care homes, nursing homes, long-term care, older people, scoping review

## Abstract

This scoping review aimed to explore the characteristics, strengths, and gaps in research conducted in Brazilian long-term care facilities (LTCFs) for older adults. Electronic searches investigating the residents (≥60 years old), their families, and the LTCF workforce in Brazil were conducted in Medline, EMBASE, LILACS, and Google Scholar, within the timescale of 1999 to 2018, limited to English, Portuguese, or Spanish. The reference lists were hand searched for additional papers. The Mixed Methods Appraisal Tool (MMAT) was used for critical appraisal of evidence. Data were reported descriptively considering the study design, using content analysis: 327 studies were included (*n* = 159 quantitative non-randomized, *n* = 82 quantitative descriptive, *n* = 67 qualitative, *n* = 11 mixed methods, *n* = 6 randomized controlled trials, and *n* = 2 translation of assessment tools). Regardless of the study design, most were conducted in a single LTCF (45.8%), in urban locations (84.3%), and in non-profit settings (38.7%). The randomized trials and descriptive studies presented the lowest methodological quality based on the MMAT. This is the first review to provide an overview of research on LTCFs for older people in Brazil. It illustrates an excess of small-scale, predominantly qualitative papers, many of which are reported in ways that do not allow the quality of the work to be assured.

## 1. Introduction

The fast growth of the older population in low- and middle-income countries [1] has allowed little time for social and health care systems to adapt. Long-term care facilities (LTCFs) are an integral part of how such systems care for older people with frailty, particularly as health conditions become more complex over time and they are no longer able to be cared for at home.

The sustainability of the LTCF sector depends upon policy and economic decisions [2]. In Brazil, where aggregate levels of wealth are lower and welfare systems are underdeveloped, the financial burden of aging is predominantly borne by families or older individuals themselves, leading to precarity of funding and lack of investment to enable development of the sector [3,4,5,6].

In 2010, there were around 3500 registered LTCFs in Brazil, and around 100,000 older people (aged 60 years and older) were living in such facilities, making the sector much smaller than in many middle- and higher-income countries [6,7]. However, estimations of the size of the sector are impaired by a lack of systems for collecting and sharing national data on LTCFs [8]. This lack of information is, in turn, a hindrance to the development of the Brazilian LTCF sector.

Research on LTCFs is an emerging field in low- and middle-income countries [9,10]. In Brazil, it has not been supported or funded in a strategic way [7,11]. This lack of co-ordination means that we are, as yet, unclear about the extent, quality, and impact of research in the sector or how it impacts on older adults’ care [7,11]. Taking stock of research carried out to date in Brazilian LTCFs will provide an understanding of the current state of the art of research in this area and highlight where work is needed.

This scoping review (SR) set out to provide an overview of the nature and extent of the scientific research conducted in Brazilian LTCFs in order to provide a summary for care providers and policymakers to inform the future endeavors in the field. The purpose of this is to give researchers, policymakers, and those commissioning research in Brazil a “big picture” overview of long-term care research conducted in Brazil over the past two decades. This overview can be used to design a coordinated plan of action for future research as well as linking to international expertise where appropriate.

We asked the following question: “What are the general features of, and gaps in, empirical research conducted across Brazilian LTCFs for those aged over 60 years?”

Our objectives were to:

1. Describe the type and quality of empirical research conducted in Brazilian LTCFs for those aged over 60 years;
2. Identify the topic areas of published research;
3. Map the regions in Brazil where this research was conducted;
4. Identify current knowledge gaps.

## 2. Methods

An international consortium established in 2019 with Brazilian, UK, and European partners (LOTUS—Improving Care in Long-Term Care Institutions in Brazil and Europe through Collaboration and Research) identified the need for this scoping review. A review protocol was previously published [11]. This paper is reported following the PRISMA Extension for Scoping Reviews (PRISMA-ScR) [12] and adheres to the theoretical framework published by the Joanna Briggs Institute to guide scoping reviews (JBI) [13].

### Eligibility Criteria

The “population–concept–context (PCC)” framework recommended by the JBI [13] was used to define the eligibility criteria for this review. Studies wholly or partly conducted in LTCFs for older people in Brazil were included if they [11]:

- Were empirical original research published in scientific journals;
- Investigated LTCF residents (older people aged 60 years and above as per/in line with the Brazilian definition of older people), their families, the LTCF workforce (e.g., healthcare professionals, care staff, and management-level staff), or LTCF organizations.

Studies involving psychiatric LTCFs, a specific category of LTCF in Brazil, were excluded (even if these included older people), as the care organization and pathways differ from non-specialist LTCFs for older people in Brazil [14].

Searches to identify relevant papers were conducted in Medline (PubMed), EMBASE (Ovid), LILACS (*Literatura Latino-americana e do Caribe em Ciências da Saúde*), and Google Scholar, from inception up to November 2018. Articles published in English, Portuguese, or Spanish were considered. No restrictions to study designs or methods were applied. The reference lists of all relevant studies were manually checked for additional eligible manuscripts.

The search strategy was developed iteratively with the input of an information scientist [11]. Significant keywords and index terms were included: “homes for the aged” (MeSH); “long-term care institutions”; “LTCF”; “geriatric long-term care facilities”; “nursing homes”; “residential facilities”; “long-term care institutions”; “long term care institutions”; “assisted living”; “old age homes”; and “Brazil” or “Brazilian”. In each block, the words were combined with the Boolean operator OR and, between the blocks, the operator AND.

After removing duplicates, two authors independently screened each article by title and abstracts against the eligibility criteria. Reference lists of included studies were also screened to identify additional relevant studies. Full-text eligible articles were each reviewed by two reviewers from a team of ten academics experienced in healthcare of older people from Brazil and the UK. When there was disagreement between them, a third reviewer reviewed the article, sighted on the area of disagreement, to achieve consensus. When the same study was reported in more than one publication, we reported the overall findings and treated them as one study.

The quality of included studies was critically appraised using the Mixed Methods Appraisal Tool (MMAT) [15]. The MMAT has different evaluation questions that enable it to be used to accommodate multiple study designs (qualitative, randomized controlled trials, non-randomized, quantitative descriptive, and mixed methods) [16]. As recommended by Hong et al. [17], the overall quality score was not calculated, and instead a more detailed presentation of the ratings of each criterion is provided.

Data were extracted by the ten authors (P.A.W., D.C.D.O., K.H.S., R.D., P.J.F.V.B., V.S., A.F.J., H.A.W.W., A.L.G., N.A.R.) independently and double-checked by three authors (P.A.W., D.C.D.O., N.A.R.) using a modified JBI data extraction tool. The following key information of each source was extracted: formal citation (author(s), title, year, institutional affiliation of the first author); region of Brazil where the study was conducted; study design by the MMAT classification; population; type of LTCF; main topics; and ethical issues. The extraction form was created and piloted by the team before the data extraction. Reasons for exclusions at each stage were registered. Study authors were contacted to request additional data if required.

Results were reported descriptively using tables, graphs, and narrative accounts using elements of content analysis in order to provide an overview of the features for the research conducted to date [13,18].

## 3. Results

### 3.1. Study Inclusion

**Figure 1:**
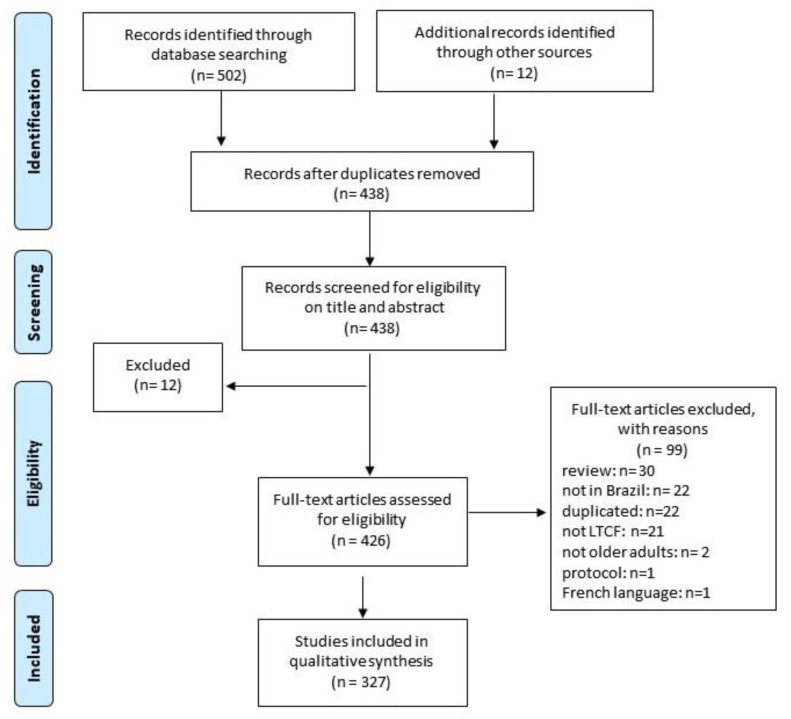
Flow chart with scoping review selection process.

A total of 512 publications were retrieved. A further 12 articles were identified during the secondary screening of the references. After deleting duplicates, 438 studies were assessed for eligibility. Ninety-nine papers were excluded, yielding 327 studies that were included. Figure 1 shows a PRISMA diagram summarizing the study selection process.

### 3.2. Features of Included Studies

Table 1 presents an overview of the included studies. Two studies are not included in the tables as they did not fit any of the designs listed on the MMAT (translation/cultural adaptation of assessment tools). Quantitative non-randomized research (QNR) (for example, non-randomized controlled trials, cohort and case–control studies, and cross-sectional analytic studies) comprised almost half of the included papers (*n* = 159; 48.9%), followed by quantitative descriptive (QD) (*n* = 82; 25.2%), qualitative (*n* = 67; 20.6%), mixed methods (*n* = 11; 3.4%), and randomized controlled trials (RCT) (*n* = 6; 1.9%).

**Figure 2:**
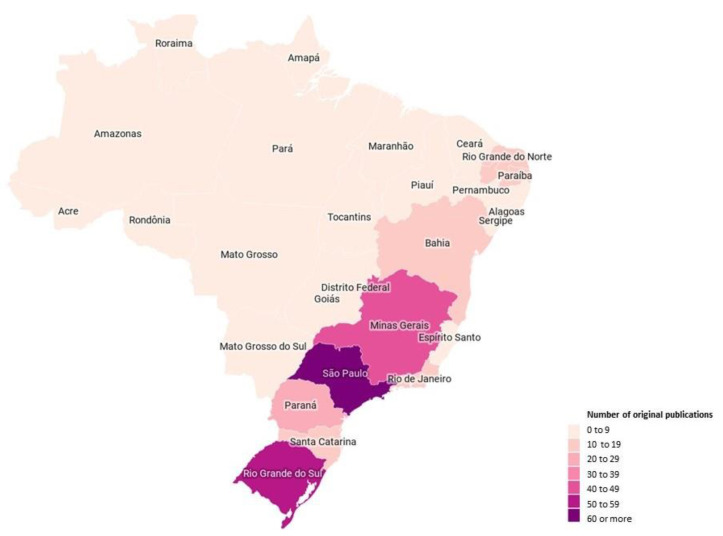
Characterization of the number of original publications included according to the Brazilian state of the institutional affiliation of the first author.

Most papers (*n* = 265; 81.5%) were published in the last ten years. The full text was available only in Portuguese in 180 publications (55.4%). Most articles had acceptable statements about ethical review; however, we could not locate any information on ethics procedures for 57 papers (17.5%). Figure 2 maps the Brazilian regions in which the studies were undertaken (according to first author institutional affiliation), illustrating the concentration of scientific research in the South and Southeast regions of Brazil.

### 3.3. Characteristics of Included LTCFs

Regardless of the study design, most were conducted in a single LTCF (*n* = 149; 45.8%), in urban locations (*n* = 274; 84.3%), and in non-profit settings (*n* = 126; 38.7%) (Table 2). A high proportion of studies failed to sufficiently report the type of setting and its location (37.0% and 38.5%, respectively). The main sample composition involved LTCF residents (*n* = 241; 74.1%) with an average of 13 older adults (2 to 59) in qualitative studies and 178 older adults (1 to 2184) in descriptive quantitative papers.

### 3.4. Research Topic Areas

The main research topics were grouped into three categories: resident outcomes (*n* = 266; 81.8%), staff and family support (*n* = 41; 12.6%), and LTCF characteristics (*n* = 18; 5.6%). Within the resident outcomes topic, the most frequent subtopics were functional capacity (*n* = 36; 13.5%), mental health (*n* = 30; 11.3%), and nutrition (*n* = 26; 9.8%). Within “staff and family support”, the main subtopics were experiences of care (*n* = 18; 43.9%) and work conditions (*n* = 4; 9.7%). Within “LTCF”, organizational context (*n* = 12; 66.6%) and policies (*n* = 6; 33.4%) were the only two subtopics. A table covering the main topic areas of research conducted in Brazilian long-term care facilities is available in the Supplementary Materials (Table S1).

### 3.5. Methodological Appraisal

Table 3 summarizes the methodological appraisal of the included articles using the MMAT. RCT and descriptive studies had a higher proportion of MMAT classified as “no” or “cannot determine” than the other designs. Therefore, the quality of the evidence based on the MMAT was lower for these designs. Studies with a qualitative design scored higher.

## 4. Discussion

This scoping review mapped the empirical scientific research undertaken in Brazilian LTCFs published in scientific journals from 1999 to 2018. We found that research in Brazilian LTCFs is in an early stage of development. From 1999—when the first study was published—until 2009, only 60 papers were reported, mostly descriptive and non-randomized quantitative manuscripts.

A recent review on global LTCF research found an increased rate of publications and citations in this field in the past 27 years, representing nearly an eightfold increase [19]. Most contributions (63%) were from the United States, Canada, and England. Brazil did not appear among the top 15 countries [19] which demonstrates that Brazil is behind the curve in terms of understanding the LTCF sector. That review [19] did not include databases beyond English ones (which may have restricted its global approach). Most publications found in our review were not published in English, so the language mismatch might have meant they were not included in prior reviews. Until such methodologies can adapt to a more international approach, the onus is therefore upon Brazilian researchers to publish in English to ensure that their data contribute to the larger debate.

The literature shows that global LTCF research has been defined by three stages: an early stage (2000–2005), where studies were primarily focused on care demand, functional, cognitive, and health status; a second stage (2006–2010), where the focus shifted to caregiving-related workforce factors; and a third stage (2011–2015), where attention moved to improving quality of care and to implementing clinical practice guidelines into LTCF homes [19]. In our review, Brazilian studies we found were mostly focused on resident outcomes and deficit-based approaches, mainly related to functional capacity, nutrition, mental health, assessment and profile, oral health, and another health status. These topics are largely related to the “early stage” focus. Research on the workforce and caregiving-related factors, person-centered care, quality of care, and quality improvements, although starting to emerge, seems relatively underdeveloped.

A large proportion of research was focused on small samples which may relate to over 20% of the papers being qualitative in design. These studies were predominantly ethnographic in nature, with limited evidence of ambition to develop middle-range or higher theory that might contribute to our understanding of Brazilian or international LTCFs, in a generalizable way. Many of the papers were outputs of research conducted towards undergraduate or postgraduate theses.

Leaving the academics to follow their muse is probably not wise. A previous review found that Italian nursing researchers, left to their own devices, tended to investigate technical and educational topics, rather than focusing on research priorities identified by the LTCF sector and care recipients [20]. One way to avoid this is to encourage and promote stakeholder participation in decisions regarding prioritization of topics for future research [10]. In such an initiative in the UK, eighty-three participants responded to a survey and ranked the five research priorities to be: questions on person-centered care, dignity, appropriate staffing, levels, and training and support requirements for LTCF staff [10].

The geographical concentration of research in the South and Southeast regions is likely to be a factor of available research funding (these regions make up 70% of the Brazilian Gross Domestic Product) [6] and also that these regions host the largest public and private universities. Based upon available data, these regions also seem to be home to the majority of LTCF homes (81.9% by one estimate) [6]. This represents a bias evident in all Brazilian research and, even more widely, reflects the concentration of public expenditure in these regions [21]. However, older people with frailty exist across Brazil and so a more disseminated approach to research is required [5]. When the United Kingdom faced similar challenges, with research concentrated in the South East around London, it overcame these challenges by developing the National Institute of Health Research (NIHR), embedded in the geographically distributed National Health Service (NHS), rather than in geographically concentrated academic institutions. This now includes a network for Enabling Research in Care Homes (EnRICH) across the country [22]. Perhaps there is something for Brazil to emulate here.

There are some limitations to our review. Despite the broad scope and a substantial number of identified publications, the searches mainly identified scientific research papers. Reports and non-peer-reviewed literature were not included. These may have included important insights into the LTCF sector more broadly. We think that it is unlikely, however, that important academic research will have been overlooked, since the pressure to publish in peer-reviewed journals is so high amongst Brazilian academics that work undertaken for government or third-sector reports is usually replicated, in some form, in the scientific literature.

## 5. Conclusions and Implications

This is the first review to provide an overview of research on LTCFs for older people in Brazil. It has drawn together almost 20 years of Brazilian-based research and illustrated an excess of small-scale, predominantly quantitative non-randomized research, many of which are reported in ways that do not allow the quality of the work to be assured. The type of research and topics researched suggest that Brazilian LTCF research is in an embryonic state—it mostly focused on resident outcomes and deficit-based approaches and was predominantly concentrated around academic institutions.

The LTCF sector in Brazil is still poorly structured and underdeveloped [5]. Recently, however, significant non-governmental initiatives such as the “Frente Nacional de Fortalecimento à ILPI (FN—ILPI)” (National Front for Strengthening the LTCF) have been developed to gather and stimulate actions to support LTCFs. These serve as democratic spaces for debates, research, planning, articulation, and promotion [23]. This paper demonstrates that even modest structured research can highlight important inequities and deficiencies in current provision in a way that can help target policy. Research on the quality of care/quality improvement, workforce, and person-centered care, for instance, seems relatively underdeveloped. There is much to be learned from initiatives undertaken to develop disseminated research networks, focusing on stakeholder priorities in a coordinated way. We contend that, as the Federal Government looks to standardize long-term care provision, it should focus on a parallel effort to establish proportionate and sustainable approaches to LTCF research.

We recommend the following actions for researchers and policymakers. The immediate priority for LTCF research in Brazil should be stakeholder consultation to define research priorities. A research agenda that reflects the priorities of stakeholders will ensure topics addressed are meaningful to the people the research is intended to serve. Stakeholder consultation needs to include a wide range of stakeholders, including older people who live in LTCFs, their relatives, care workers, practitioners, management staff, and professional organizations relevant to the Brazilian LTCF context (FN—ILPI; Brazilian Society of Geriatrics and Gerontology). Due to the diversity across Brazilian states, it will be important to consult in a geographically inclusive way, recruiting stakeholders across different states. Setting research priorities will enable researchers and decision-makers in funding organizations to make informed choices around where research efforts should be placed. The focus then should be on developing a small number of sufficiently funded, high-quality research projects to investigate these. Lessons can be learned from how other countries have structured long-term care research. International knowledge exchange and sharing and collaboration will therefore be valuable. There is evidence of international knowledge exchange and sharing currently taking place. Jacinto et al. [7] outlined topics for research into Brazilian LTCFs which were identified during an international workshop which took place in Brazil in 2019. The workshop was supported by academics from across Brazil, the UK, the Netherlands, and Austria. An important metric will be the number of Brazilian LTCF publications accepted to international journals and thus contributing to the broader debate of what good LTCFs look like.

## Figures and Tables

**Table 1:** Characteristics of included studies regarding primary research conducted in Brazilian long-term care facilities (LTCFs) published in scientific journals by methodology.

	Qualitative (*n* = 67)	Descriptive (*n* = 82)	Non-Randomized (*n* = 159)	RCT (*n* = 6)	Mixed Methods (*n* = 11)
Publication Date					
1999–2009	11 (16.4%)	19 (23.1%)	24 (15.1%)	1 (16.6%)	5 (45.5%)
2010–2015	42 (62.6%)	45 (54.9%)	83 (52.2%)	1 (16.6%)	5 (45.5%)
≥2016	14 (20.9%)	18 (21.9%)	52 (32.7%)	4 (66.8%)	1 (9.0%)
Language					
English	6 (8.9%)	15 (18.3%)	47 (29.5%)	1 (16.6%)	2 (18.2%)
Portuguese	46 (68.7%)	51 (62.2%)	73 (45.9%)	3 (50.0%)	7 (63.6%)
At least Portuguese/English	15 (22.4%)	16 (19.5%)	39 (24.6%)	2 (33.4%)	2 (18.2%)
Geographic area *					
North	1 (1.5%)	0	4 (2.5%)	0	0
Northeast	13 (19.4%)	21 (25.6%)	32 (20.1%)	1 (16.6%)	0
South	29 (43.2%)	17 (20.7%)	35 (22.0%)	3 (50.0%)	7 (63.6%)
Southeast	14 (20.9%)	34 (41.5%)	65 (40.9%)	0	4 (36.4%)
Midwest	4 (5.9%)	6 (7.3%)	16 (10.0%)	1 (16.6%)	0
≥2 geographic area	3 (4.5%)	2 (2.4%)	3 (1.9%)	0	0
NR	3 (4.5%)	2 (2.4%)	4 (2.5%)	1 (16.6%)	0
1st Author Institution					
Public University	44 (65.7%)	59 (71.9%)	106 (66.7%)	4 (66.8%)	6 (54.5%)
Private University	19 (28.3%)	17 (20.7%)	33 (20.7%)	2 (33.2%)	5 (45.5%)
Health Service	2 (3.0%)	2 (2.4%)	6 (3.8%)	0	0
Governmental Agency	0	1 (1.2%)	1 (0.6%)	0	0
Others	2 (3.0%)	2 (2.4%)	1 (0.6%)	0	0
NR	0	1 (1.2%)	2 (1.2%)	0	0
Ethical approval ^†^					
Yes	59 (88.0%)	64 (78.0%)	132 (83.0%)	5 (83.4%)	8 (72.7%)
NR	8 (12.0%)	18 (22.0%)	27 (17.0%)	1 (16.6%)	3 (27.3%)

RCT: randomized controlled trial; NR: not reported; * the Federal Constitution of 1988 divides Brazil into five regions: North, Northeast, Midwest, Southeast, and South; ^†^ ethical approval was clearly informed by the authors.

**Table 2:** Characteristics of the long-term care facilities (LTCFs) studied in the included papers from primary research conducted in Brazilian LTCFs published in scientific journals by the type of methodology.

	Qualitative (*n* = 67)	Descriptive (*n* = 82)	Non-Randomized (*n* = 159)	RCT (*n* = 6)	Mixed Methods (*n* = 11)
Type of setting					
Profit	2 (3.0%)	0	1 (0.6%)	0	0
Non-profit	**32 (47.7%)**	**31 (37.8%)**	59 (37.1%)	**3 (50.0%)**	1 (9.0%)
Both	12 (17.9%)	17 (20.7%)	36 (22.6%)	0	**5 (45.5%)**
NR	21 (31.4%)	**34 (41.5%)**	**63 (39.6%)**	**3 (50.0%)**	**5 (45.5%)**
Setting Location					
Rural	1 (1.5%)	0	1 (0.6%)	0	0
Urban	**43 (64.2%)**	**42 (51.2%)**	**94 (59.1%)**	2 (33.2%)	**8 (72.7%)**
Both	0	5 (6.1%)	8 (5.0%)	0	0
NR	23 (34.3%)	35 (42.7%)	56 (35.3%)	**4 (66.8%)**	3 (27.2%)
Number of LTCF					
1	**46 (68.7%)**	**35 (42.1%)**	**60 (37.7%)**	**3 (50.0%)**	**5 (45.5%)**
2–5	9 (13.4%)	19 (22.9%)	37 (23.2%)	**3 (50.0%)**	1 (9.0%)
6–10	8 (11.9%)	12 (15.6%)	25 (15.7%)	0	0
≥11	3 (4.5%)	08 (9.7%)	22 (13.8%)	0	4 (36.5%)
NR/NA	1 (1.5%)	08 (9.7%)	15 (9.4%)	0	1 (9.0%)
(Min–Max, mean, median)	(0–52, 3.7, 1)	(1–156, 10.1, 2)	(1–125,6.4, 2)	(1–5, 2.0, 1.5)	(1–52, 14.4, 1)
Sample composition					
Older adults	**33 (49.2%)**	**64 (78.0%)**	**133 (83.6%)**	**6 (100%)**	**5 (45.5%)**
Total (Min–Max, mean, median)	Total = 428 (2–59, 12.9, 10)	Total = 11,358 (1–2184, 177.4, 76)	Total = 22.747 (4–3903, 171.0, 81.0)	Total = 164 (13–37, 27.3, 30)	Total = 204 (8–55, 40.8, 43)
Family	1 (1.5%)	0	0	0	0
Total (Min–Max, mean, median)	Total = 6				
Staff	19 (28.3%)	7 (8.5%)	7 (4.4%)	0	3 (27.2%)
Total (Min–Max, mean, median)	Total = 337 (7–40, 17.7, 16)	Total = 411 (12–181, 58.7, 38.5)	Total = 459 (22–181, 65.5, 45)		Total = 281 (38–181, 93.6, 62)
LTCF characteristics	3 (4.4%)	7 (8.5%)	2 (1.3%)	0	0
Total (Min–Max, mean, median)	Total = 59 (1–52, 19.6, 6)	199 (4–156, 28.4, 7.5)	Total = 80 (29–51, 40.0, 40)		
Managers and stakeholders	3 (4.4%)	1 (1.2%)	0	0	
Total (Min–Max, mean, median)	Total = 18 (5–7, 6.0, 6)	Total = 67			
Older adults × Non-institutionalized older adults	0	2 (2.4%)	15 (9.4%)	0	1 (9.0%)
Total (Min–Max, mean, median)		Total = 192 (15–177, 96.0, 96) × Total = 273 (30–243, 136.5, 136.5)	Total = 1180 (14–393, 78.7, 42) × Total = 16,839 (14–598, 112.6, 76)		Total = 30 × Total = 30
Older adults × Staff	2 (3.0%)	1 (1.2%)	2 (1.3%)	0	2 (18.3%)
Total (Min–Max, mean, median)	Total = 13 (3–10, 6.5, 6.5) × Total = 25 (9–16, 12.5, 12.5)	Total = 62 × Total = 33	Total = 57 (11–46, 28.5,28.5) × Total = 40 (15–25, 20.0, 20)		Total = 314 (6–308,157.0, 157) × Total = 50 (7–43, 25.0, 25.0)
Older adults × Family	1 (1.5%)				
Total (Min–Max, mean, median)	Total = 3 × Total = 3				
Older adults × Managers	3 (4.4%)				
Total (Min–Max, mean, median)	Total = 27 (8–11, 13.5, 8) × Total = 17 (3–7, 8.5, 7)				
Family × Staff	1 (1.5%)				
Total (Min–Max, mean, median)	Total = 13 × Total = 19				
Managers × Staff	1 (1.5%)				
Total (Min–Max, mean, median)	Total = 20 × Total = 36				

NR: not reported; NA: not applicable; LTCFs: long-term care facilities. The numbers in bold represents the most frequent values.

**Table 3:** Critical appraisal of included sources of evidence through the Mixed Methods Appraisal Tool (MMAT), *n* = 325.

Screening Questions (for All Types)						Qualitative (*n* = 67)														
Are there clear research questions?			Do the collected data allow to address the research questions?			Is the qualitative approach appropriate to answer the research question?			Are the qualitative data collection methods adequate to address the research question?			Are the findings adequately derived from the data?			Is the interpretation of results sufficiently substantiated by data?			Is there coherence between qualitative data sources, collection, analysis, and interpretation?		
Y	N	C	Y	N	C	Y	N	C	Y	N	C	Y	N	C	Y	N	C	Y	N	C
62	5	-	53	9	5	56	6	5	38	7	22	40	3	24	37	6	24	37	11	19
						**Quantitative randomized controlled trials (*n* = 6)**														
						Is randomization appropriately performed?			Are the groups comparable at baseline?			Are there complete outcome data?			Are outcome assessors blinded to the intervention provided?			Did the participants adhere to the assigned intervention?		
Y	N	C	Y	N	C	Y	N	C	Y	N	C	Y	N	C	Y	N	C	Y	N	C
6	-	-	5	-	1	1	1	4	4	-	2	3	2	1	2	3	1	2	1	3
						**Quantitative non- randomized (*n =* 159)**														
						Are the participants representative of the target population?			Are measurements appropriate regarding both the outcome and intervention (or exposure)?			Are there complete outcome data?			Are the confounders accounted for in the design and analysis?			During the study period, is the intervention administered (or exposure occurred) as intended?		
Y	N	C	Y	N	C	Y	N	C	Y	N	C	Y	N	C	Y	N	C	Y	N	C
156	3	-	140	6	13	57	47	55	120	19	20	126	7	26	56	58	45	120	15	24
						**Quantitative descriptive (*n =* 82)**														
						Is the sampling strategy relevant to address the research question?			Is the sample representative of the target population?			Are the measurements appropriate?			Is the risk of nonresponse bias low?			Is the statistical analysis appropriate to answer the research question?		
Y	N	C	Y	N	C	Y	N	C	Y	N	C	Y	N	C	Y	N	C	Y	N	C
78	3	1	64	11	7	37	21	24	32	31	19	61	9	12	29	12	41	58	8	16
						**Mixed methods (*n* = 11)**														
						Is there an adequate rationale for using a mixed methods design to address the research question?			Are the different components of the study effectively integrated to answer the research question?			Are the outputs of the integration of qualitative and quantitative components adequately interpreted?			Are divergences and inconsistencies between quantitative and qualitative results adequately addressed?			Do the different components of the study adhere to the quality criteria of each tradition of the methods involved?		
Y	N	C	Y	N	C	Y	N	C	Y	N	C	Y	N	C	Y	N	C	Y	N	C
11	-	-	7	1	3	7	1	3	6	4	1	6	4	1	3	2	6	6	4	1
	Category with most of the studies with YES																			
	Category with most of the studies with NO																			
	Category with most of the studies with CANNOT DETERMINE																			

## Data Availability

Dataset is publicly available on: Wachholz, Patrick Alexander; Oliveira, Deborah; Hinsliff-Smith, Kathryn; Devi, Reena; Villas Boas, Paulo José Fortes; Shepherd, Victoria; Jacinto, Alessandro Ferrari; Watanabe, Helena Akemi Wada; Gordon, Adam Lee; Ricci, Natalia Aquaroni, 2021, “RESEARCH ON LONG-TERM CARE FACILITIES FOR OLDER PEOPLE IN BRAZIL FROM 1999–2018”, https://doi.org/10.7910/DVN/TWQ9JL, Harvard Dataverse, V1.

## Supplementary Materials

The following are available online at https://www.mdpi.com/1660-4601/18/4/1522/s1, Table S1: Main topic areas of scientific peer-reviewed research conducted in Brazilian long-term care facilities, grouped into three categories.
