# Supplementary material for: Mapping Research Conducted on Long-Term Care Facilities for Older People in Brazil: A Scoping Review
Source: Int J Environ Res Public Health. 2021 Feb 5;18(4):1522. doi: 10.3390/ijerph18041522 (PMC7915754; doi:10.3390/ijerph18041522)
Supplement: Supplementary file 1 [file ijerph-18-01522-s001.pdf]

Table S1. Main topics areas of scientific peer-reviewed research conducted in Brazilian long-term care facilities, grouped into three categories.

Resident Outcomes	Total *n*	Staff and Family Support	Total *n*	LTCF Characteristics	Total *n*
Functional capacity	36	Views about care	18	Organizational context	12
Mental Health	30	Family functionality	6	Policies	6
Nutrition	26	Work condition	4		
Balance/Falls/Fractures	22	Occupational health	3		
Pharmacotherapy	21	Views about patients' conditions	2		
Perceptions/Experiences	19	End of life	2		
Oral Health	17	Staff profile	2		
Assessment/ Profile	17	Work condition	1		
Frailty	10	Spirituality	1		
Reasons for LTCF admission	9	Occupational health	1		
Infection	8	Social support	1		
Urinary Incontinence	7				
Quality of life	6				
Ulcers	4				
Pain	4				
Leasure Time/Recreational activities	4				
Dizziness/Hearing	3				
Signs	3				
Spirituality	3				
Other health conditions	3				
Communication	3				
Nursing Diagnosis	3				
Sleep	2				
Smoke	2				
Immunity	1				
Mortality	1				
Hospital admission	1				
End of life	1				

LTCF: long-term care facilities.
